# Domestication effects on crowing in chickens: variation between wild and captive red junglefowl and domestic white Leghorn and the genetic architecture of crowing vocalizations

**DOI:** 10.1098/rstb.2024.0199

**Published:** 2025-05-15

**Authors:** Dominic Wright, Jennie Westander, P. Jensen

**Affiliations:** ^1^ IFM Biology, Linköping University, Linköping, Sweden; ^2^ Skansen Foundation, Department of Zoology, Stockholm, Sweden; ^3^ Department of Zoology, Stockholm University, Stockholm, Sweden

**Keywords:** domestication, quantitative trait locus, crowing

## Abstract

The crowing of the male chicken is a charismatic example of vocal display in a bird. It is regarded as the main territorial announcement of the ancestral red junglefowl. The call has been preserved throughout domestication, although several of its elements have been altered. To assess these alterations, we assayed crowing spectrograms from wild and captive-held red junglefowl populations from India, along with two red junglefowl populations held in long-term captivity in Sweden, and a domestic white Leghorn breed. We find consistent differences between the different Indian red junglefowl and the domestic white Leghorn for a range of characteristics, including the duration of the last syllable and the number of formants and their frequency in the last and second-to-last syllable. To analyse the genetic architecture of crowing vocalization, we performed a quantitative trait loci (QTL) experiment using a wild × domestic advanced intercross to identify QTL that explained a large percentage of the variation present for the duration of the last syllable and the number of formants in the second to last syllable. With this study we thus demonstrate consistent differences in red junglefowl and white Leghorn chickens and identify a relatively simple genetic architecture for some of these traits.

This article is part of the theme issue ‘Unravelling domestication: multi-disciplinary perspectives on human and non-human relationships in the past, present and future’.

## 1. Introduction

The crowing of the male chicken, the cockerel, is a charismatic example of vocal display in a bird. It is regarded as the main territorial announcement of the ancestral red junglefowl (RJF) [1], at the same time being a reliable indicator of the status, size and health of the emitter [2]. The call type has been preserved throughout domestication, although several of its elements have been altered [3]. There are not a wide range of studies describing the ontogenesis of crowing, but those studies that do exist show that the essential patterns of each individual are innate, though some modifications can occur due to social rank (and possibly some other features) [2]. These modifications are, however, minimal. It appears that the main indicator of social rank is the frequency with which a male crows, not the composition of the actual call [2]. This is in direct contrast to songbird vocalizations, which have a strong learned component [4].

RJF, ancestors of present-day chickens, inhabit large regions of southeast Asia where they were most likely first domesticated about 8000 years ago [5,6] (although a recent analysis claims a considerably later time point for first domestication [7]). The wild ancestor lives in stable social groups based on a tight family structure and a group typically holds a small territory centred around a roosting tree and contains several males and females and their offspring [8,9]. The territory borders are defended vigorously against intruders mainly by the males and are announced by means of loud crowing, emitted mainly during dawn and dusk [10].

Chickens were originally thought to be domesticated primarily for sport and leisure, based on rich archaeological findings of remains indicating that staged cockfighting was common in the societies where domestic fowl first appeared [10]. Such activities profited from the strong territoriality of the males, including their ability to crow. Crowing is also used for communication within the group, being a reliable indicator of the strength and health status of the emitter [2]. Within a group, there is strong sexual selection related to crowing, since females preferentially mate with healthy and dominant males with large combs and profound crowing abilities [11].

Whereas crowing stands out as a spectacular chicken vocalization, it is by no means the only vocal utterance of the species. In fact, the chicken has one of the most versatile vocal repertoires among birds, comprising a large set of sounds used in different communication contexts [1]. For example, different vocalizations are used to warn group members against predators depending on whether they approach on the ground or in the air, and particular sounds are used to communicate discoveries of food items by the hen when calling her chicks. The intensity of each vocalization type can also be varied extensively to communicate, for example, the degree of danger from a predator or the quality of a food source, with a profound exception: the crowing.

Unlike all other chicken vocalizations, crowing is highly stereotyped, with large inter-individual variation but high intra-individual consistency [12]. It consists of three or four syllables, of which the last is extended, giving rise to the traditional English rendition ‘cock-ka-doodle-dooo’ [1]. The composition of the call is consistent within individuals, with respect to fundamental frequencies, formants and durations of each syllable, while there can be considerable variation between males and, in particular, between different genetic strains [12]. This suggests that variations in the performance of crowing have a strong genetic component, with the strong repeatability of this trait being an upper limit to heritability [12].

Domestication, the process where populations become genetically adapted to a life among humans, is known to produce a suite of physiological and other phenotypical changes, referred to as the ‘domestication syndrome’ [13,14]. This includes, for example, changes in age at sexual maturity, coloration, size, fearfulness and brain composition. Even if modifications in vocalizations are usually not included in the domestication syndrome, it is clear that many domesticates show altered vocal behaviour. For example, domesticated Bengalese finches have a more complex song than their wild ancestors [15,16], and domesticated cavies (guinea pigs) differ from their wild counterparts both in vocal repertoire and the structure of different vocalizations [17]. Dogs have a considerably modified vocal repertoire compared to ancestral wolves [18], and foxes selected for increased tameness (as a model of domestication) use a different set of vocalizations both towards conspecifics and humans compared to unselected individuals [19,20].

During chicken domestication, crowing appears to have been particularly targeted for selection throughout history, and there is a large breed variation in different aspects of this vocalization [12]. Some breeds are specifically bred for the ability to produce long crowing, and e.g. males of the Japanese breed Naganakidori can produce crows that each last for more than 15 s [3]. This is profoundly different from the typically abrupt truncated call of the RJF [1]. Hence, crowing offers an interesting possibility to dissect the genetic underpinnings of a domestication-related trait in chickens.

Previously, we have used quantitative trait locus (QTL) analysis to analyse the genetic basis for a number of traits modified during chicken domestication. For example, we have localized strong genetic candidates for changes in social behaviour, fear and stress, pigmentation and brain composition [21–32]. This has been possible by utilizing a unique intercross between RJF and a domesticated laying hen, providing a powerful population for genetic mapping of domestication-related traits.

Here, we use an extensive sample of crowings, recorded from (i) wild RJF in their natural geographical range, (ii) captive bred populations of the same species and (iii) a domesticated white Leghorn breed to describe intra- and inter-specific variation, as well as individual consistency and modifications to different vocal parameters caused by domestication. We then analyse crowing in the previously mentioned wild × domestic intercross population and use these data to map the genetic architecture of crowing vocalization. This allows a deeper genetic analysis of a hitherto largely under-investigated aspect of the domestication syndrome.

## 2. Material and methods

### (a) Study populations

#### (i) Wild and park crowing vocalizations

Recordings of crowing from wild and captive RJF males were obtained during a four month field trip to the Himachal Pradesh region in northern India. Four different locations containing wild birds (Barog, Banon, Ghanahatti and Renuka) and five wildlife parks (Renuka, Khariun, Kufri, Shimla and Delhi) were included in the sampling from India. In each of the wildlife parks, small groups of RJF were kept in aviaries. In the four other places, recordings were made in the natural habitat of the birds or from overnight lodges in the early mornings around daybreak and around dusk.

Crowings were recorded on an opportunistic sampling scheme, with the attempts to obtain as many calls as possible from as many males as possible. Due to the large individual differences in fundamental frequencies and durations of the crowings, in most instances, it was possible to distinguish between calls from different males even when they were not within sight and thereby to maximize the number of individuals' samples at every site. However, in the case of the populations from Banon and Ghanahatti, this was not possible, and therefore all vocalizations were grouped together for these populations. Where it was possible, in the parks or in the wild, the averages of each trait were taken for each individual before then calculating the population metrics. An average of 6.4 recordings were made per individual. Recordings were made with a microphone and recorder (Panasonic VDR-D50) at distances varying between about 5 and 100 m. The recordings were taken during a five month stay in Himachal Pradesh, northern India. A variety of locales throughout the region were visited based on the likelihood of RJF being present, and birds were sampled if found. For the captive birds, no written information was present regarding their background in terms of the number of generations in captivity, though given the proximity of RJF in the area, it seems unlikely to be many. All aviaries for these Indian captive birds were naturally furnished aviaries, i.e. natural foliage, perches and refuges were all present. The size of these aviaries was a minimum of 20 m^2^, with one (Shimla) up to around 200 m^2^. Typically, around 10 birds were present per aviary (with more females than males), though 20−30 were present in Shimla (also female biased).

In addition, crowing vocalizations were also recorded from a captive population of RJF derived from a Thai zoo (Götala_LiU) that has subsequently been maintained at Linköping University field station over many generations [33], and a population derived originally from Copenhagen zoo (Cph_LiU), also maintained at the same place. These different RJF crowing vocalizations were then compared with a laboratory-held population of domesticated White Leghorn (White Leghorn LiU) also maintained at the Linköping University field station [33]. In the case of these laboratory-housed populations, recordings were obtained opportunistically from a distance of 1−5 m when the birds were present in their home pens. We used a Yamaha Pocketrak 2G microphone and recorder and filtered out a total of 27 unique males through a combination of listening for specific characteristics and inspecting spectrograms.

In the case of the different populations housed in Sweden, the Götala RJF population was originally obtained from Frösö zoo in 1999, where they had been maintained since 1993. Prior to that, they were imported from Thailand from a semi-wild population. For the Copenhagen RJF population, they were imported to the Linköping facility in 2004 from Copenhagen zoo. Prior to this, the population had been free-roaming in the zoo since the 1950s. The white Leghorn strain of layers was first maintained at the Swedish Land Agricultural University from the 1970s onwards, before being moved to Linköping in the 1990s. They are referred to as the L13 strain of white Leghorn.

#### (ii) Wild × domestic intercross population

In the same way as for the pure domestic and wild lines described above (those maintained at Linköping University field station), we recorded calls from a total of 95 unique males of an intercross between the two lines. This intercross was originally set up by crossing one RJF male with four white Leghorn chickens and then proceeding with intercrossing in subsequent generations (see [21,34] for a detailed description of the intercross breeding). The RJF male originally came from a captive Thai zoo population (and was therefore a separate population from the two RJF populations measured above), while the white Leghorns were a line that has been maintained in Sweden since the 1970s and referred to as the L13 population (see above). This population was included as the comparison domestic white Leghorn in the population comparison, while in the case of the Thai RJF, only the founding individual was available, so this particular population was unavailable for further testing. The males used in the present study were from the eighth advanced intercross generation, bred and maintained in the same experimental unit as the pure domestic and wild lines described above. For these laboratory (LiU) housed birds (these being the Copenhagen_RJF, the Götala_RJF, the WL-L13 and the F8 intercross birds), these were all maintained in the same conditions and densities (maximum of 50 per pen). Birds were housed in a 4 m × 4 m × 4 m multilevel enclosure with perches and nest boxes available, in single-sex groups as adults but in mixed-sex groups as chicks and juveniles.

### (b) Spectrogram analysis of the calls

A specific vocalization is characterized by a fundamental frequency, which defines the pitch of the sound. In addition, each vocalization has a range of spectral maxima, which are referred to as formants. These are caused by acoustic resonance in the vocal apparatus, and the number of formants can differ substantially between different sounds. They are normally distinct multiples of the fundamental frequency (which is normally the lowest frequency band in a spectrogram). One can say that the number of formants and the relative strength of each is what gives the sound its specific quality. For example, if a flute and a violin both play the same tone, say C, the reason that you can hear which one is the flute is that the instruments create different numbers of formants; this is also why you can hear the difference between when Jussi Björling and Luciano Pavarotti sing, even if they sing the same tune. Formants are visible in the spectrogram as local maxima (darker stripes) parallel to the fundamental tone at distinct frequency distances from the fundamental frequency, and you can therefore easily count the number of formants in any spectrogram.

**Figure 1.**
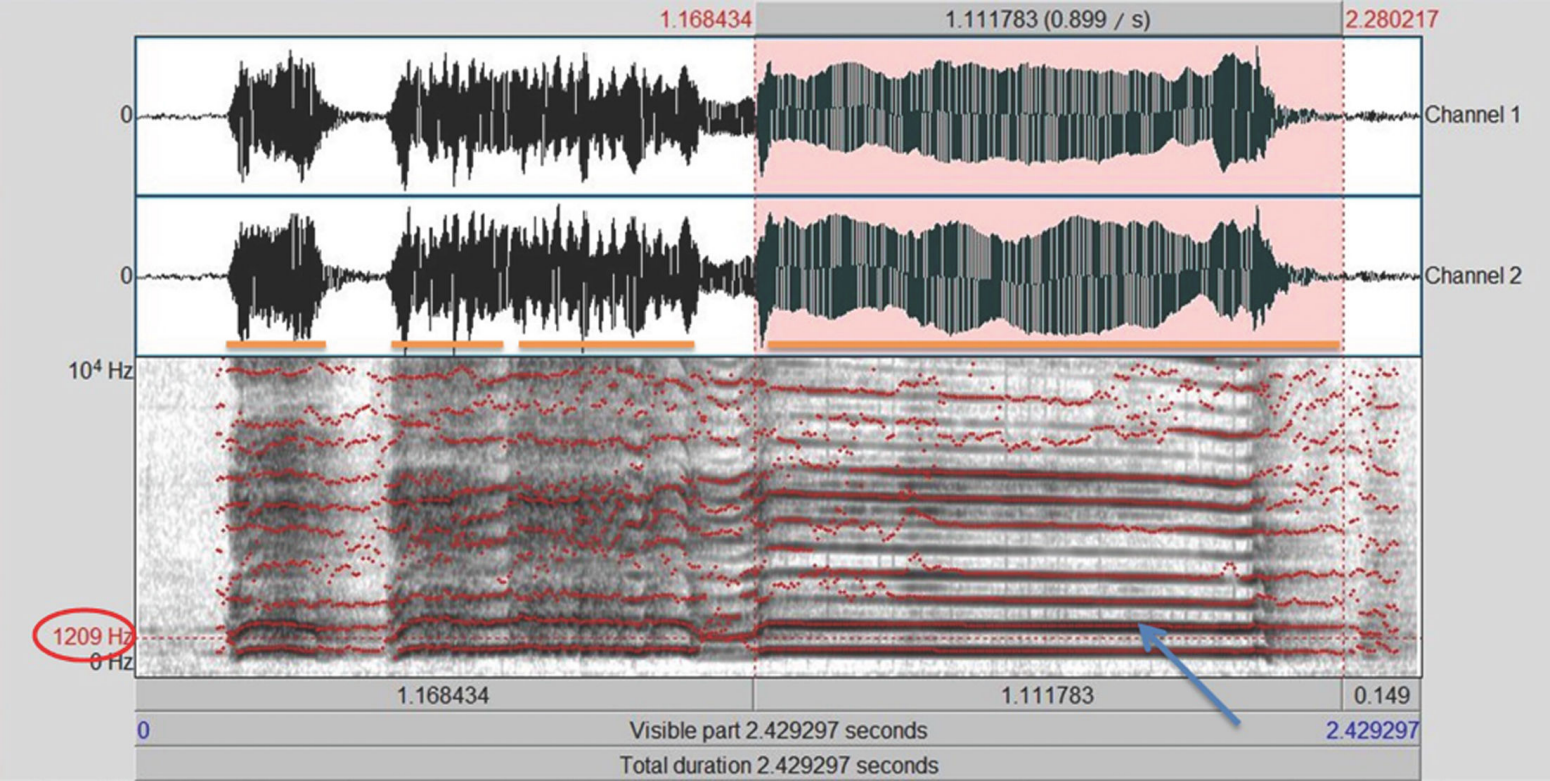
Breakdown of crowing vocalization measurements. This sample figure illustrates the phenotypes measured from each vocalization. Orange lines are used to mark the number of syllables present in each (in this illustration, four syllables are present). The duration of the last syllable is marked in pink and measured in seconds. The number of formants of the last syllable is indicated with a blue arrow, with this arrow indicating one of the formants. The same measurement is also applied to the second-to-last syllable. The most distinct formant of the last and second-to-last syllables is calculated in Hertz and is calculated using the darkest (most prominent) average trace in the particular syllable (in this case, 1200 Hz).

Each call from each of the studies was represented as a spectrogram using the software Praat v. 5.3.29 [35] (see figure 1 for a breakdown of these measurements). From each spectrogram, we assessed the number of syllables and the duration of the last syllable (the ‘tail’ part of the crow), as well as the second-to-last syllable. Furthermore, we measured the number of formants in each of these two syllables, as well as the most distinct formant of the last and second-to-last syllable. These give the frequency of these syllables in Hertz.

Differences between the populations were tested by firstly grouping the populations into four different categories: wild (RJF that were free-living), captive RJF in India, captive RJF in Sweden, and domestic white Leghorn in Sweden. Traits were then analysed using a linear model regressing the trait on category, with a Tukey pairwise comparison test used to assess pairwise differences between the different categories.

### (c) DNA extraction and QTL analysis

DNA preparation was performed by Agowa GmbH (Berlin, Germany), using a standard salt extraction technique [36] on whole blood. A total of 652 single nucleotide polymorphism markers were used to generate a map of length approximately 92 675 cM, with an average marker spacing of approximately 16 cM. Additional details of marker generation, map generation and the like can be found in Johnsson *et al.* [21]. QTL analysis was performed using R/qtl [37] for both standard interval mapping and epistatic analyses. Interval mapping was performed using an additive + dominance model. Map generation and permutation threshold measures were performed using the F_8_ dataset to account for the map expansion from the F_2_ to the F_8_. In the QTL analysis, batch and bodyweight were initially included in the model as fixed effects, while a principal component analysis (PCA) was used to account for population structure (see below), with the principal components included as a covariate. Non-significant terms were then dropped from the final model.

A PCA approach was used to control for any potential family structure [38]. In this case, family structure refers to when later generations of the advanced intercross start diverging from the expected 1 : 2 : 1 homozygote–heterozygote–homozygote marker segregation ratio, leading to an excess of homozygote classes, and hence potentially different relatedness between individuals, as compared to the F_2_ population. This occurs when too many of the individuals come from the same combination of individuals from the previous generation. Although we maximized this as much as possible, with 52 families used to produce the F_8_ individuals, we also used the PCA approach in case any residual substructure was present. We calculated the first 10 PCs based on the full genotype data, then tested these for significance in each vocalization QTL regression. All significant PCs were then retained in the final model. This approach allowed us to both control for population substructure and also test for epistatic interactions. In addition, where only one offspring is taken from a particular pairing, then no population substructure should occur [39]. In the case of the 95 F_8_ individuals used here, these came from 52 different family pairings, greatly reducing the family substructure just by the experimental design. Two locus (digenic) epistatic analysis was performed as per the guidelines given in the R/qtl handbook [40]. A global model incorporated standard main effects, and epistasis was built up starting with the most significant loci and working down for each trait.

### (d) Significance thresholds

Significance thresholds for all QTL analysis were calculated using permutation tests [41,42]. A suggestive significance level of a genome-wide 20% threshold was used (due to this being more conservative than the standard suggestive threshold [43]). The approximate significant threshold was a log of the odds (LOD) score of approximately 5.5, while the suggestive threshold was approximately 4.5. Confidence intervals (CI) for each QTL were calculated with a 1.8 LOD drop method (i.e. where the LOD score on either side of the peak decreases by 1.8 LOD) [44]. The nearest marker to this 1.8 LOD decrease was then used to give the CI in megabases. Epistatic interactions were also assessed using permutation thresholds generated using R/qtl, once again with a 20% suggestive and 5% significant genome-wide threshold used (using the guidelines given in [40]). In all cases, 1000 permutations were used to calculate the thresholds.

## 3. Results

### (a) Call structure differences between domestic and wild chickens

Marked differences in the duration of the last syllable and the number of formants in the last syllable and second-to-last syllable were observed between the different Indian-based RJF populations on one hand (both wild and those present in parks) and the domestic white Leghorn breed sampled (see table 1 for pairwise comparisons and table 2 for individual population characteristics). Longer last syllable durations and more formants in the last and second-to-last syllables were present in the domestic white Leghorn breed, as compared to the RJF. However, a distinction was also found between crowing characteristics between the captive RJF populations present in Scandinavia (Copenhagen and Götala populations) and the wild and captive RJF populations present in India. In fact, one of the Scandinavian populations had longer last syllable duration and greater formants in the last and second-to-last syllables as compared to the white Leghorn (though not significantly so). Tukey pairwise comparisons are given in table 1.

**Table 1.** First half: Tukey post hoc significance values of two-way comparisons for crowing vocalization traits in wild and domestic (WL) birds. For the comparisons, ‘Wild_RJF’ refers to Banon, Barog, Ghanahatti and wild-living Renuka populations; ‘India_Park_RJF’ refers to Shimla, Khariun, Kufri and Reunka park-living RJF based in India; ‘LiU_RJF’ refers to Götala and Copenhagen long-term captive populations housed in Sweden; ‘WL_LiU’ refers to the white Leghorn L13 line housed in Sweden. Second half (*t*-test comparisons): pairwise two-sample *t*-test comparisons of the three LiU (captive) populations with each other.

comparison	last_syllable_length	#_formants_in_last_syllable	#_formants_in_second_last_syllable	freq_most_distinct_formant_last_syllable (Hz)	freq_most_distinct_formant_second_last_syllable (Hz)
India_park_RJF vs Wild_RJF	0.99 NS	0.000***	0.000***	0.559 NS	0.864 NS
LiU_RJF vs Wild_RJF	0.000***	0.000***	0.000***	0.267 NS	0.000***
WL vs Wild_RJF	0.000***	0.000***	0.000***	0.000***	0.000***
LiU_RJF vs Indian_Park_RJF	0.000***	0.000***	0.120 NS	0.053 NS	0.000***
WL vs Indian_Par_RJF	0.000***	0.000***	0.730 NS	0.000***	0.000***
WL vs LiU_RJF	0.666 NS	0.711 NS	0.60 NS	0.054 NS	0.000***
***t*** **‐test comparisons**					
WL_LiU vs Götala_LiU	0.07	0.36 NS	0.49 NS	0.04*	0.03*
WL_LiU vs Cph_LiU	0.78 NS	0.65 NS	0.61 NS	0.002**	0.0002**
Götala_LiU vs Cph_LiU	0.07	0.92 NS	0.92 NS	0.42 NS	0.23 NS

**Table 2.** Population characteristics of the different wild RJF (Barog_wild, Banon_wild, Ghanahatti_wild, Renuka_wild), RJF in Indian parks (Shimla_park, Khariun_park, Kufri_park, Renuka_park, Delhi_park) and long-captive European-held RJF (Cph_LiU, Götala_LiU), as well as a domestic WL population (White Leghorn_LiU). The average scores of the Indian Park RJF, Wild RJF and LiU RJF are also given. The number of individuals in each group is given (n), as well as the mean and standard deviation of the different crowing vocalization characteristics. These are the duration (in seconds) of the last syllable of the crow, the number of syllables in the crow, the number of formants in the last syllable, the number of formants in the second-to-last syllable, the frequency (in Hz) of the most distinct formant in the last syllable and the frequency (in Hz) of the most distinct formant in the second-to-last syllable.

		last_syllable_length (s)		number_syllables		#_formants_in_last_syllable		#_formants_in_second_last_syllable		freq_most_distinct_formant_last_syllable (Hz)		freq_most_distinct_formant_second_last_syllable (Hz)	
population	n	mean	s.d.	mean	s.d.	mean	s.d.	mean	s.d.	mean	s.d.	mean	s.d.
Barog_wild	2	0.17	0.03	4	0.00	3.72	1.81	4.40	1.98	1974	155	2588	28
Banon_wild	*	0.14	0.03	4	0.00	1.38	0.74	2.13	0.35	2100	102	2680	42
Ghanahatti_wild	*	0.28	0.12	4	0.00	2.09	0.29	2.05	0.21	2122	64	2509	114
Renuka_wild	3	0.30	0.04	4	0.00	2.27	0.46	2.23	0.25	2426	203	2885	27
Shimla_park	3	0.29	0.07	4	0.00	2.71	0.55	3.97	2.00	1987	37	2480	133
Khariun_park	2	0.22	0.00	4	0.00	5.08	0.12	5.92	0.35	2344	287	2630	93
Kufri_park	2	0.20	0.01	4	1.41	3.00	1.41	4.70	1.56	3172	822	2346	185
Renuka_park	1	0.24	0.00	4	0.00	3.70	0.00	6.70	0.00	1874	0	2643	0
Delhi_park	5	0.25	0.05	4	0.00	5.28	2.33	6.39	0.98	2051	296	2607	132
Götala_LiU	8	0.92	0.25	4	0.00	7.78	1.25	6.68	1.72	1979	225	2175	288
Cph_LiU	5	0.74	0.20	4	0.00	7.72	1.94	6.49	1.73	1999	92	2298	124
White_Leghorn_LiU	14	0.77	0.27	4	0.39	7.25	1.28	6.01	1.52	1737	60	1823	103
F8 AIL	95	0.96	0.24	4	0.67	6.94	1.66	5.73	1.75	2013	382	2014	314
India_Park_RJF	*	0.24	0.03	4	0.28	3.95	0.88	5.53	0.98	2286	288	2541	109
Wild_RJF	*	0.22	0.05	4	0.00	2.36	0.83	2.70	0.70	2156	131	2665	53
LiU_RJF	*	0.83	0.22	4	0.00	7.75	1.59	6.58	1.73	1989	158	2236	206

### (b) Genetic architecture of the last syllable and number of formants

**Figure 2.**
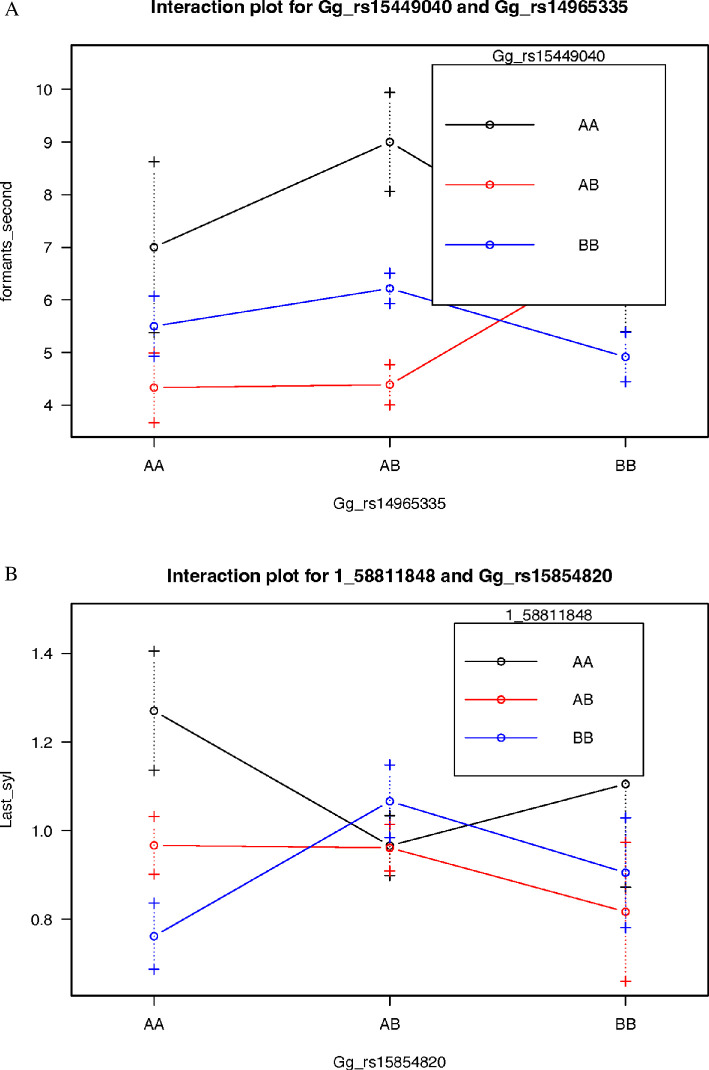
(A) Epistatic interaction for last syllable duration QTL 1@791 and 7@173. AA, RJF genotype; AB, heterozygote genotype; BB, WL genotype. (B) Epistatic interaction for last syllable duration number of formants in second-to-last syllable QTL 3@668 and 11@104.

A total of three QTL were detected for the duration of the last syllable (see table 3). Of these, two were an epistatic pair, while one was a single locus effect. In both the case of the major effect of the epistatic interaction and the single locus effect, both show that the QTL have a greater effect in the RJF allele (see figure 2). Hence, the RJF alleles confer an increase to last syllable length, rather than a decrease. The three QTL explained a total of 82% of the total variation present in the intercross birds assayed. Although these QTL appear to be transgressive (i.e. the alleles displaying a greater effect on the phenotype derived from the opposite parental strain than expected), this may not be the case when we consider that the intercross was between a Thai RJF and the L13 white Leghorn, with the syllable duration and number of formants unknown for this RJF population.

**Table 3.** QTL identified in the wild × domestic advanced intercross. Chromosome, position in cM, LOD square, % effect size (*r*^2^), additive effect and its standard error (negative values indicate the greater effect allele is derived from the RF genotype, positive values that the greater effect allele is derived from the WL genotype), dominance effect and its standard error, and the confidence interval obtained using a 1.8 LOD drop.

trait	chr	position	LOD	*r* ^2^	add	s.e.	dom	s.e.	interaction	CI lower	CI upper	lower marker	upper marker
last_syllable_duration	1	791	14.5	0.33	−1.13	4.25	−1.57	5.11	1@792: 7@173	777	803	Gg_rs13652874	Gg_rs14832321
last_syllable_duration	7	173	14.1	0.32	0.90	3.62	−0.96	3.62	1@792:7@173	167	175	RBL4833	Gg_rs14614489
last_syllable_duration	17	51	7.5	0.14	−0.06	0.03	−0.24	0.06		36	70	Gg_rs15033588	Gg_rs13744523
#_formants_second_last_syllable	14	9	6.2	0.09	−0.64	0.22	1.34	0.28		2	15	Gg_rs13530101	Gg_rs13530937
#_formants_second_last_syllable	3	668	15.8	0.29	−1.41	0.29	−2.35	0.38	3@668.0:11@104.0	662	674	3_103455805	3_108000014
#_formants_second_last_syllable	11	104	12.9	0.22	−0.09	0.24	0.00	0.33	3@668.0:11@104.0	96	104	Gg_rs14025092	RBL3647
#_formants_second_last_syllable	12	71	5.7	0.08	−0.87	0.24	0.95	0.41		62	127	Gg_rs13609494	Gg_rs14979113

The only other trait where significant QTL were detected was for the number of formants in the second-to-last syllable. Four QTL were detected for this trait, with these explaining 81% of the total variation present in the intercross. All of these QTL once again displayed a greater effect being derived from the RJF alleles (see table 3).

### (c) Overlaps between vocalization QTL and brain composition and behavioural QTL

The intercross used for this study has also been used to map a variety of other traits, including brain composition and anxiety behaviour. These traits were selected as they have the potential to be linked to vocalization behaviour. In the case of anxiety behaviour, three separate tests were performed: an open field test (measuring movement performed in a novel arena), a social reinstatement test (measuring exploration in an arena with conspecifics at one end) and a tonic immobility test (length of time spent in a state of tonic immobility when induced). For brain composition, these were both proportional and relative sizes of the cerebellum, optic lobes, cerebrum and midbrain. When considering the QTL detected in these previous analyses, three different QTL detected in the social reinstatement assay overlapped with the QTL detected in this study, as well as one QTL for relative cerebellum size (see table 4).

**Table 4.** Behavioural and brain composition QTL that overlap the vocalization QTL identified in the wild × domestic advanced intercross. ‘SR’ in the trait refers to the social reinstatement test, and the QTL describes the length of time animals spent in the start zone away from the conspecifics, the length of time in the stimulus zone near to the conspecifics (average of two trials), and the longest time spent adjacent to the conspecifics of the two tests performed. Chromosome, position in cM, LOD score, effect size (*r*^2^), additive effect and its standard error (negative values indicate the greater effect allele is derived from the RF genotype, positive values indicate the greater effect allele is derived from the WL genotype) and the confidence interval obtained using a 1.8 LOD drop are also provided.

trait	chr	position (cM)	lower CI	upper CI	LOD	*r* ^2^	add	s.e.
SR_maximum_time_in_start_zone	1	803	776	834	5.6	0.036	−0.35	0.09
SR_latency_to_enter_stimulus_zone_average	7	175.5	159	194	8.5	0.055	−15.27	7.39
SR_maximum_latency_to_enter_stimulus_zone	7	175.5	173	197	8.9	0.057	−12.83	7.79
relative cerebellum size	7	159	150	171	7.4	0.044	4.24	6.47

## 4. Discussion

Initially, we find a clear difference between the calls of the wild RJF and the domesticated WL, and also differences when comparing long-term captive RJF and wild or newly captured RJF. Last syllable length, number of formants in the last syllable and the frequencies of the last and second-to-last formants were all different between the RJF and the domestic WL-L13 line. The domestic WL birds display a longer last syllable with more formants in it and lower frequencies of the last syllable. However, when we separate the wild RJF and the Indian RJF in parks with the two RJF populations that have been maintained for many generations in captivity, a slightly different picture emerges. In this case, the last syllable and number of formants in the last syllable are both longer in the two long-captive RJF populations (Götala and Copenhagen). In fact, for these two traits, the long-captive populations are either similar to or even greater than the domestic WL population.

Crowing vocalizations have been shown to be a selectable trait in chickens, with a wide degree of variation present between domestic breeds [12]. In particular, long crowing breeds are found in Asia, notably in Indonesia [45] and Japan [3]. These birds are indigenous breeds and have been hypothesized to be derived from fighting cocks, based on mitochondrial D-loops from a limited number of Japanese breeds [3], though other authors have questioned this interpretation [46]. The long crowing characteristics are considered desirable [47] and appear to have been selected upon [47]. Even now, crowing contests are held in Germany, Indonesia and Japan (e.g. http://longcrowers.de/). Long crowing breeds can crow for up to 60 s, making them outliers in crowing vocalizations in domestic breeds. These responses to selection are borne out by what we see in the differences between wild RJF and the domestic WL in this study. We demonstrate that crowing characteristics differ markedly between wild and domestic birds. Not only that, but we demonstrate that crowing vocalizations also differ between wild RJF populations. In addition to the variation between wild RJF, the differences between wild RJF and the two RJF populations that were long captive and maintained in Europe could indicate either inadvertent selection occurring during captivity, introgression from domestic birds into these RJF populations at some point during captivity, or that these long-captive RJF populations simply came from different wild populations with their own variation in crowing characteristics.

The variation in crowing characteristics present in the wild populations also indicates that considerable inter-population variation is present for these traits. Given this variation, it would suggest at least some of these crowing sub-traits are not under very strong directional selection to allow such variation to persist or that local selection can select for different trait values in different populations. In addition, the QTL that were detected had a relatively large effect size (though are likely over-estimates, see below), though these of course represent a different type of inter-population variation (namely, the difference between domestic and wild phenotypes). The fact that the intercross represents wild × domestic inter-populations does mean that the genetic architecture could be very different when comparing inter-population variation between different wild populations, and most likely is, especially if there is any strong selection (that would then rapidly fix the large effect loci). Furthermore, the inter-population differences in the different wild RJF populations mean that quite a wide degree of wild variation exists, and therefore different genetic architecture may be revealed when different wild × domestic intercrosses are used. In this case, some of the observed differences may not actually be related to domestication but from differences in the true wild progenitor of the domestic bird and the RJF population used for the intercross.

The fact that the advanced intercross was started with only one RJF male (with origins from Thailand) has some caveats associated with it. Most notably, it was unclear to which sub-species this individual belonged. Two sub-species are associated with Thailand: *G. g. gallus* and *G. g. spadiceus* [48]. It has been shown that these two sub-species are the closest to the domestic breeds of chickens (only 0.5–3% sequence divergence), though with introgressions from other RJF sub-species [48]. Therefore, that the RJF used was Thai indicates it should be a good model of the RJF progenitor, but a larger population of these birds would have allowed a full phenotypic assessment to be made and also the assessment of the potential for domestic introgression, which is always an issue with any wild RJF population.

When analysing the genetic architecture of the different components of the crowing vocalization, we identify loci (both epistatic and fixed) for three of the traits. In the case of two of these traits (last syllable and number of formants in the second-to-last syllable), we identify three and five loci, respectively. All of these QTL display the greater effect (i.e. the alleles that confer the greatest effect on the phenotype) coming from the RJF allele (i.e. the RJF alleles confer a longer last syllable and a greater number of formants in the second-to-last syllable). Although these appear to be transgressive when comparing the wild-sampled RJF to the WL, this may not necessarily be the case. The intercross used to map these traits (WL-L13 × Thai RJF) used a different RJF population as compared to the two captive RJF populations we tested. When we look at the tested RJF populations, one of them (Götala) possessed a longer mean last syllable duration and more formants in the second-to-last syllable, as compared to the white Leghorn domestic population. It is therefore possible that the Thai population had a phenotype closer to the Götala RJF, although with no more of these specific Thai RJF birds available, it is impossible to verify. Given this, it remains to be confirmed as to whether the loci that we have identified in this specific intercross will also be the same loci that regulate the differences between domestic birds and the wild RJF that were also sampled directly from India.

Of the loci detected, these initially appear to explain a large amount of the total variation present in the intercross—79% of the last syllable duration, 74% of the number of formants in the second-to-last syllable, and 73% of the variation in the frequency of the formants in the last syllable. However, due to the Beavis effect [49], it is probable that the effect sizes of the detected QTL are over-estimated. However, even with this over-estimation, a sizeable proportion of the variation is detected, which would imply a relatively simple genetic architecture, and certainly that these vocalization traits have a strong genetic component to them. This relatively simple genetic architecture would appear to confirm the increased crowing duration in domestic birds is genetic in origin, with the caveat that we do not know how representative our intercross is of wild × domestic comparisons in general for this particular phenotype. The high amount of variance explained by the QTL detected would also indicate a very high heritability (at least in this specific intercross), and the large effects would imply a rapid response to selection.

Studies assessing the genetic basis of quantitative vocalizations are rather under-studied. Despite vocal communication being important in a huge range of different species and for a wide variety of different functions, few studies go into detail as to the genetic basis of such traits. However, one of the most studied aspects of vocalizations to date is infant vocalization, particularly in mice [50–53]. There are differences in infant vocalizations between mouse breeds that have enabled the mapping of QTL related to infant ultrasonic vocalizations [54]. Interestingly, when examining the heritabilities of sub-traits relating to ultrasonic vocalizations, the estimates for the number and frequency of calls were low (implying strong selection), whereas the estimates for the duration and amplitude of calls were high, with syllable length being placed in this category [54]. In this regard, these traits appear similar to the crowing vocalizations in chickens, with our study implying high heritability (and selectability) for the duration of the last syllable in crowing, as well as composition.

Gene knockouts and pharmacological interventions have also been shown to modify vocalizations. For example, pharmacological blockage of central NK_1_ receptors in both mouse and guinea pig pups was found to attenuate neonatal vocalizations [55], indicating that these receptors are involved in the regulation of neonatal vocalizations and could also be used in the treatment of mood and anxiety disorders [55]. An autism gene knockout model of the post-synaptic scaffolding proteins ProSAP1/Shank2 was shown to modify ultrasonic vocalization in both adult and pup mice in both a quantitative and qualitative manner [56]. In relation to these gene knockouts, and in particular with regard to anxiety behaviours, these seem to suggest more the presence or absence of calling, rather than variation in call structure, would be related to anxiety, though quantitative differences were also observed.

The wild × domestic advanced intercross used in this study has also been assayed for a wide variety of other traits, with the respective QTL also mapped, including for a variety of anxiety-related behaviours (open field behaviour [25], social reinstatement behaviour [27] and tonic immobility [29]), as well as brain composition [24] and predictability in behavioural responses [31]. By checking for overlaps between the QTL for these different traits with those detected for vocalization, it is possible to detect potential trade-offs arising from either pleiotropy or linkage to the loci of interest [22,57]. In the case of the vocalization QTL detected here, we find overlaps with three separate anxiety-related social reinstatement QTL and one QTL affecting the relative size of the cerebellum. The fact that we see overlaps with anxiety-related traits is of some note, in particular when considering the murine QTL detected for subsonic vocalizations and their relationship with anxiety traits (see above). Similarly, the overlap with the QTL for relative cerebellum size could potentially indicate a role for this tissue in the regulation of crowing vocalizations in the chicken, with the cerebellum itself proportionally expanding in domestic chickens [24,58]. However, when we consider the direction of effect of these brain and anxiety-related QTL, the most anxious genotypes are RJF in origin, while for the cerebellum QTL, the WL genotype showed the largest increase. This is opposite to the crowing vocalization QTL we find here in that the RJF gives longer last syllable length. This would suggest that these are therefore linked, rather than pleiotropic, effects; i.e. these loci do not control multiple aspects of the domestication phenotype, but represent multiple loci that are genetically linked. Furthermore, the differing directions also suggest that these are not representative of any specific trade-offs, at least in the intercross studied here.

This overlap between vocalization, anxiety behaviour and brain composition could be related to the formation of the genetic architecture of domestication, with these linked loci forming discrete clusters throughout the genome. Evidence for this effect has already been identified in this intercross [21–23,27,57,59–61]. For example, clusters containing QTL for comb size, fecundity, bone allocation and egg production [21–23,59] loci were found to co-localize in discrete clusters, with several other examples for other traits also identified [25,27]. In this regard, if the vocalization QTL are another such example, they may not reflect a trade-off at the genetic level (i.e. that the loci for the regulation of inter-population variation in crowing occur in the cerebellum), but rather that they represent a more disparate selection of domestication traits that just happen to have been fixed in a domestication cluster. The only way to confirm this is to identify the actual loci (or potentially just the causal genes) for vocalization QTL, and then assay them in specific sections of the brain to see in exactly which tissues the causal genes are expressed.

In conclusion, we demonstrate that a clear difference between wild RJF and domestic chickens is the composition of their crowing vocalization, and this has a strong genetic component. Wild RJF also exhibit variation in crowing characteristics between different populations, and we have characterized what we believe to be the largest number of wild and wild-caught RJF vocalizations here. By performing a QTL mapping experiment, we find a relatively simple genetic architecture for this trait, and in particular for the duration of the last syllable.

## Data Availability

Data is provided as electronic supplementary material [62].
